# Recurrent Perihepatic Abscesses Arising from a Gastric Remnant Leak: Delayed Complication of a Revision Roux-en-Y Gastric Bypass

**DOI:** 10.1155/2021/5510526

**Published:** 2021-02-27

**Authors:** Aboubacar Kaba, Florence-Damilola Odufalu, Zarir Ahmed, Charlene Prather, Mustafa Nazzal

**Affiliations:** ^1^Saint Louis University School of Medicine, St. Louis, MO, USA; ^2^Department of Internal Medicine, Saint Louis University, St. Louis, Missouri, USA; ^3^Division of Gastroenterology, Department of Internal Medicine, Saint Louis University, St. Louis, Missouri, USA; ^4^Division of Transplant Surgery, Department of Surgery, Saint Louis University, St. Louis, MO, USA

## Abstract

Roux-en-Y gastric bypass is a procedure commonly used for weight loss associated with improved outcomes and decreased complications when compared to some counterparts. The procedure involves restriction of the stomach that is achieved by creation of a gastric pouch and bypass of the duodenum and a portion of the jejunum to aid in restrictive and malabsorptive weight loss. While many complications, both early and late, have been described following the procedure, recurrent perihepatic abscess has not been described in the literature. We present a case of a 66-year-old woman with recurrent extrahepatic abscesses following revision of a Roux-en-Y gastric bypass.

## 1. Introduction

Roux-en-Y gastric bypass (RYGB) is a commonly performed weight loss procedure and is often preferred over other options such as adjustable gastric banding, duodenal switch, and vagal stimulation. RYGB is both restrictive and malabsorptive and thus has its advantages in increased, more durable weight loss with less morbidity and mortality than its counterparts [1–3]. While there are many complications of Roux-en-Y gastric bypass surgery, perihepatic abscess from a poorly drained enteroatmospheric fistula formation is a rare complication that has not been extensively reviewed in the literature. We report a case of recurrent perihepatic abscess in a patient with a history of RYGB.

## 2. Case Description

**Figure 1:**
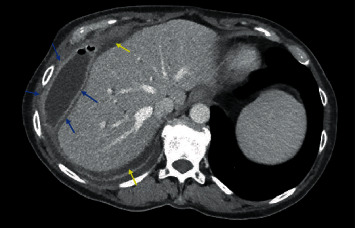
11 cm gas and fluid collection between the liver and diaphragm consistent with abscess (blue arrows) with perihepatic free fluid (yellow arrows).

**Figure 2:**
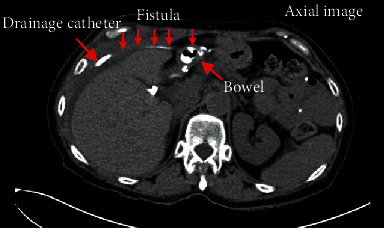
CT fistulogram showing an enterprising-atmospheric fistula with gastric remnant. Figure provided by Louis Maurice Morel-Ovalle, MD, DABR, and Raj Bant.

**Figure 3:**
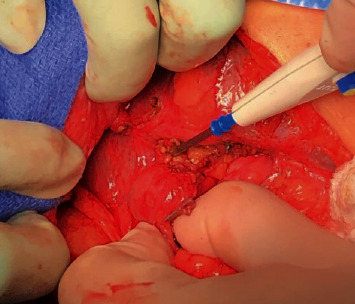
Exploratory laparotomy revealing gastric remnant and fistula at the left hepatic lobe.

A 66-year-old woman with a history of morbid obesity status post-Roux-en-Y gastric bypass (RYGB) in 2003 complicated by gastrogastric fistula requiring revision with partial gastrectomy of remnant stomach with revision of gastrojejunostomy (GJ) in 2017 presented to her primary care provider with worsening abdominal pain, fevers, chills, and anorexia for 2 weeks. She was instructed to present to the emergency department (ED) for further evaluation. Since her RYGB revision, she had several hospital admissions for recurrent polymicrobial perihepatic abscesses, beginning 5 months after her revision, requiring prolonged courses of IV antibiotics and abscess drainage, however, with no overt cause identified. On exam at the ED, vital signs were notable for a temperature of 101.1 F, blood pressure of 101/55, and a heart rate of 69 BPM after receiving IV fluids and morphine for pain control. Pertinent laboratory assessment revealed alkaline phosphatase (ALP) of 280 U/L, alanine transaminase (ALT) of 83 U/L, aspartate aminotransferase (AST) of 208 U/L, and WBC of 11.5 × 10^9^/L with 80% neutrophils. Abdominal computed tomography (CT) scan revealed an 11 cm extrahepatic abscess between the liver and diaphragm and adjacent right pleural effusion (Figure 1). She was started on broad-spectrum antibiotics and had a CT-guided abscess drain placement, returning 150 mL of green/yellow purulent fluid with subsequent cultures grown Klebsiella pneumoniae and Citrobacter freundii. Following the intervention, she was monitored in the inpatient setting and her clinical picture began to improve. However, due to the recurrent nature of the patient's abscess, the differential for the cause of her presentation was expanded to include fistula, biliary leak, or retained surgical product. An upper endoscopy was performed to assess postsurgical anatomy which revealed Roux-en-Y gastrojejunostomy anatomy with gastrojejunal anastomosis with healthy mucosa and a normal jejunum. A hepatobiliary iminodiacetic acid (HIDA) scan was completed to assess for biliary leak which revealed grossly normal hepatobiliary scan with no abnormal tracer accumulation in the right upper quadrant. An early CT fistulogram was completed which identified the known perihepatic collection but did not demonstrate a new fistulous tract. A repeat CT fistulogram showed a fistulous communication to the biliopancreatic limb (Figure 2). However, due to the limitations of the CT fistulogram, suspicion remained for a fistula in a portion of the bypass not adequately visualized. Thus, the patient was discharged on antibiotic therapy with her drain in place and follow-up scheduled with hepatobiliary surgery. She underwent an exploratory laparotomy with findings of a fistulous tract between the gastric remnant staple line and abdominal wall (Figure 3). A portion of the gastric remnant was adherent to the left lobe of the liver (Figure 3). There were no signs of distal obstruction or malignancy that could have contributed to the formation of the fistulous tract. The staple line of the gastric remnant along with the fistula was resected and oversewn in 2 layers with successful repair. Additionally, the right lobe of the liver was mobilized and no apparent abscess formation was noted. Her postoperative course was complicated by surgical site infection and was managed with local wound care. In the 10 months following her exploratory laparotomy, she has had no further recurrences of perihepatic abscesses.

## 3. Discussion

The general surgeon must be prepared to diagnose and treat the many serious complications that can result from bariatric surgery [4]. Postoperative complications of RYGB can be divided into early and late. Early complications, defined as those which occur within 30 days of the operation, include fistula, bowel obstruction, and pulmonary embolism [5]. Late complications include strictures, enteric fistulas, cholelithiasis, marginal ulcers, ventral and internal hernias leading to small bowel obstruction, dumping syndrome, gastric remnant distension, and nutritional deficiencies [6, 7]. Notably, patients with complications particularly in the gastric remnant present physicians with both diagnostic and therapeutic dilemmas. Feared complications in the gastric remnant include peptic ulcer disease, gastric remnant bleeding, malignancy, volvulus, choledocholithiasis, obstruction, and fistula formation with the gastric remnant or adjacent structures, as previously seen in our patient [8]. However, perihepatic abscess from enteric fistula formation in the gastric remnant is a rare complication that is challenging to diagnose. This challenge is due to the length of the Roux limb limiting endoscopic approaches, insufficient insufflation of the bypassed stomach limiting interventional radiology approaches, and insufficient reflux of contrast limiting contrast studies [9, 10].

While liver abscesses are the most common visceral abscess, only 5-15% of liver abscesses are reported from surgical complications [11]. To date, there are no other reported cases of recurrent perihepatic abscesses from a gastric fistula following revision of a RYGB. The etiology of recurrent perihepatic abscesses in our patient resulted from fistula formation at the gastric staple line leading to an exposure to enteric secretions. The patient had five life-threatening infections and several hospitalizations related to a fistulous tract which was not identified endoscopically or radiographically, initially. However, during her final admission, a repeat fistulogram identified a fistulous communication to her biliopancreatic limb. Following an exploratory laparotomy to correct the fistula, her symptoms resolved and she has not had further recurrences.

This case highlights the need for providers to continue to have a high index of suspicion of pathology occurring in the gastric remnant and to consider fistula formation in the gastric remnant, as these can be difficult to see with advanced imaging modalities. However, due to the rarity of severe delayed complications of the remnant, benefits of maintaining the gastric remnant for nutritional value, lack of evidence to support the need for routine follow-up of the remnant, and the favorable success rate of the Roux-en-Y procedure, it currently remains a suitable option for bariatric surgery.

## Data Availability

No data were used to support this study.
